# Efficacy of Proanthocyanidins in Nonsurgical Periodontal Therapy

**DOI:** 10.1016/j.identj.2022.08.006

**Published:** 2022-09-24

**Authors:** Evelina Alkimavičienė, Rasa Pušinskaitė, Nomeda Basevičienė, Rasa Banienė, Nijolė Savickienė, Ingrida Marija Pacauskienė

**Affiliations:** a Department of Dental and Oral Pathology, Lithuanian University of Health Sciences, Kaunas, Lithuania; b Department of Biochemistry, Medical Academy, Lithuanian University of Health Sciences, Kaunas, Lithuania; c Department of Pharmacognosy, Lithuanian University of Health Sciences, Kaunas, Lithuania

**Keywords:** Periodontitis, Proanthocyanidins, Periodontal debridement, Randomised controlled trial

## Abstract

**Background:**

The aim of this work was to evaluate the efficacy of proanthocyanidins (PACNs) as an adjunctive periodontal therapy in patients with periodontitis.

**Methods:**

Patients with periodontitis (stage III–IV) were included in this randomised clinical study. Patients with periodontitis received 2 different treatment modalities: minimally invasive nonsurgical therapy only (MINST group) or minimally invasive nonsurgical therapy and subgingival application of collagen hydrogels with PACNs (MINST + PACNs group). Clinical periodontal parameters, that is, pocket probing depth (PPD), clinical attachment level (CAL), bleeding on probing (BOP), plaque index (PI), were evaluated before treatment and after 8 weeks. Concentrations of immunologic markers, matrix metalloproteinase-3 (MMP-3), and tissue inhibitor of matrix metalloproteinase-1 (TIMP-1) in saliva were assessed at baseline and at 8-week follow-up.

**Results:**

Forty-six patients diagnosed with periodontitis were randomised into 2 groups: 23 patients in the MINST group and 23 patients in the MINST + PACNs group received the intended treatment. PACNs combined with MINST resulted in additional statistically significant PPD reduction and CAL gain in moderate periodontal pockets by 0.5 mm (*P* < .05) on average compared to MINST alone. Additional use of PACNs did not result in additional statistically significant improvement of BOP or PI values. Application of PACNs showed significant reduction of MMP-3 levels in saliva after 8 weeks (*P* < .05).

**Conclusions:**

Adjunctive use of PACNs in MINST resulted in better clinical outcomes for moderate pockets. Additional use of PACNs improved MMP-3 concentration in saliva more than MINST alone. Biochemical analysis revealed that MMP-3 concentration in saliva reflected the periodontal health state.

## Introduction

Periodontitis is a chronic infection that leads to gingival inflammation, alveolar bone resorption, gingival attachment loss, and tooth loss.^1^ Many studies report on the changes in inflammatory biomarkers in saliva or gingival crevicular fluid of patients with periodontitis and on the use of these findings in disease diagnosis and further treatment.^2^^,^^3^ Specifically, biochemical analysis of salivary samples revealed a wide variety of inflammatory mediators, which include metalloproteinases (MMPs) and tissue inhibitors of MMPs (TIMPs).^4^ MMP and TIMP levels reflect the periodontal health: Higher levels of MMPs and TIMPs are found in saliva or gingival crevicular fluid of patients with nontreated chronic periodontal disease than in samples of patients with healthy periodontal conditions or treated periodontal disease.^5^^,^^6^

Different variations of nonsurgical periodontal therapy, such as minimally invasive nonsurgical therapy (MINST), conventional one-stage or partial quadrant scaling and root planing, and one-stage full mouth oral disinfection, demonstrate successful treatment outcomes.^7^, ^8^, ^9^, ^10^, ^11^ A minimally invasive treatment strategy is the new standard in modern periodontology. The term “minimally invasive nonsurgical therapy” includes the careful scaling and root planing procedure with the use of ultrasonic scalers with thin tips and mini- and micro-curettes and magnification loupes, creating a minimal wound and effectively removing subgingival deposits.^12^ Conventional nonsurgical periodontal treatment includes standard tips together with ultrasonic devices and Gracey curettes for subgingival debridement. The MINST procedure was chosen instead of conventional nonsurgical periodontal treatment in the protocol for the current trial for more accurate elimination of the biofilm from deep periodontal pockets. Nonsurgical periodontal therapy combined with adjunctive treatment (eg, chlorhexidine chips, local and systemic antibiotics, probiotic lozenges) shows additional improvements on clinical findings.^13^, ^14^, ^15^, ^16^

Proanthocyanidins (PACNs) are oligomers or polymers of monomeric flavan-3-ols produced as an end product of the flavonoid biosynthetic pathway.^17^ PACNs are found in diverse structures of plants (leaves, flowers, fruits, or seeds). Research has shown that PACNs demonstrate antioxidative, anticancerous, anti-inflammatory, antimicrobial activities in different fields of medicine.^18^ Recently, PACNs were proposed as a viable adjunct to periodontal treatment.^19^ Preclinical studies have shown high antibacterial and anti-inflammatory capacities of PACNs, which could reduce periodontal inflammation and promote periodontal tissue regeneration.^20^ In addition, PACNs demonstrate a specific antibacterial characteristic to attack periodontopathogenic bacteria (*Porphyromonas gingivalis*) but save the oral commensal bacteria (*Streptococcus salivarius*).^21^^,^^22^

Collagen hydrogels are widely used as active ingredient carriers in pharmacology.^23^^,^^24^ In addition, research suggests the use of collagen hydrogel as a scaffold for periodontal tissue regeneration.^25^^,^^26^ Non–commercially available type I collagen hydrogels were manufactured for research purposes in the current study.

To the authors’ knowledge, no clinical study has been done before to evaluate the effects of locally derived PACNs on nonsurgical therapy of periodontitis. The aim of the current clinical study was to assess the effects of collagen hydrogels with PACNs from *Pelargonium sidoides* DC root extract (a detailed protocol for PACN sample preparation according to a previous study^20^) as an adjunct to MINST in patients with generalised periodontitis. In addition, biochemical analysis of salivary biomarkers (MMP-3 and endogenous TIMP-1) was performed. The null hypothesis of no statistically significant differences in mean pocket probing depth (PPD) changes in moderate pockets from baseline to 8 weeks between test (MINST + PACNs) and control (MINST) groups was tested.

## Materials and methods

### Trial design

We conducted a randomised clinical trial in the Department of Dental and Oral Pathology at the Lithuanian University of Health Sciences in Kaunas, Lithuania, from January to May 2019. Systemically healthy individuals with periodontitis were involved in the study. All included patients must have signed an informed consent form, were willing and able to attend follow-up appointments, and agreed to coded data collection. Patients were given enough time to analyse the protocol of the study and were free to exit the study at any time without a specific reason. After they signed the informed consent form, they were randomly assigned to the control or experimental group (allocation ratio 1:1).

The study was performed according to CONSORT guidelines for randomised controlled clinical trials.^27^ This trial was registered on 20 August 2021 (NCT05015387). Ethical permission was issued by the Regional Biomedical Research Ethics Committee (No. BE-2-38). Detailed data supporting the present study may be obtained upon reasonable request.

### Participants

Male and female patients visiting the Department of Dental and Oral Pathology at the Lithuanian University of Health Sciences in Kaunas, Lithuania, were considered for enrollment.

Patients with periodontitis (stage III–IV) were included in the study according to the 2017 World Workshop on the Classification of Periodontal and Peri‐Implant Diseases and Conditions.^28^ The inclusion criteria in treatment groups were as follows:

• Patients with stage III and IV periodontitis with slow or moderate rate of progression (A/B) (radiographic bone loss extending to middle or apical third of the root, tooth loss due to periodontitis, maximum probing depth ≥6 mm, horizontal and vertical [≥3 mm] bone loss)
• Systemically healthy individuals
• Those with ≥20 remaining teeth
• Patients aged 30 years or older

Patients were excluded if they:

• Had stage I and II periodontitis (radiographic bone loss in coronal third, no tooth loss due to periodontitis, maximum probing depth ≤5 mm, mostly horizontal bone loss)
• Had systemic disease(s)
• Had antibiotic therapy during the last 3 months
• Had periodontal treatment during the last 6 months
• Were pregnant or lactating
• Claimed to be allergic to the adjunct (PACNs)

### Intervention

Patients with stage III and IV periodontitis were allocated by the same examiner (RP) to 2 groups: The first group received MINST only (MINST group), and the second group received MINST and single-time subgingival application of collagen hydrogels with PACNs (MINST + PACNs group). Non–commercially available type I collagen hydrogels were manufactured for the research purposes of the current study.

Prior to the clinical trial, patients received intraoral cavity preparation (emergency dental treatment, hopeless teeth extraction). Standardisation of the clinical trial was achieved by professional oral hygiene and individual oral hygiene instructions for each patient. During the 8-week term of the trial, the patients were informed not to use other chemical control materials.

The clinical trial protocol included several appointments. At baseline, all patients received a periodontal examination, and saliva samples were collected. Samples of unstimulated saliva were collected with 3 sterile cotton rolls deposited for 5 minutes in the oral cavity and stored in sterile tubes at −80 °C. Both treatment groups underwent a full-mouth MINST procedure by a single periodontist (EA). The procedure was performed in a sterile field. Under local anesthesia, the participants underwent MINST with thin and delicate tips together with ultrasonic (Acteon Satelec Suprasson P5 Booster) and hand instruments (Gracey micro-curettes [Hu-Friedy] and mini-curettes [LM-Dental]) and magnification of 4.5×. During the MINST procedure, the periodontist was not informed about the patients’ group assignment (MINST or MINST + PACNs). After the procedure, patients’ group allocation was revealed to the periodontist (EA) by the examiner (RP) and, accordingly, each patient either received adjunctive treatment (the MINST + PACNs group received collagen hydrogel chips with PACNs placed subgingivally in periodontal pockets with PPD ≥4 mm) or had the procedure ended without adjunctive therapy (MINST group). Postoperative care instructions were given.

Patients returned for a follow-up appointment 8 weeks after baseline. Periodontal reevaluation of encoded patients was performed by a single examiner (EA). Unstimulated saliva samples were collected in the same manner as mentioned earlier.

### Outcomes

For data analysis, pockets were assigned into 2 categories: moderate (PPD, 4–6 mm) and deep (PPD ≥7 mm).^14^^,^^29^ The primary outcome variable was the mean PPD change from baseline in moderate sites at 8 weeks. Secondary outcome variables were the mean PPD change from baseline in deep sites at 8 weeks; the mean clinical attachment level (CAL), bleeding on probing (BOP), and plaque index (PI) changes from baseline in moderate as well as deep sites at 8 weeks; and the mean MMP-3 and TIMP-1 changes from baseline at 8 weeks.

Examiner calibration was performed with the use of the Cohen's kappa coefficient (≥0.85). Ten patients with more than 5 teeth with PPD and CAL *≥*5 mm were selected and examined twice (at a 15-minute interval between the first and second measurements) with a universal periodontal probe (PCPUNC156; Hu-Friedy).

### Immunologic investigation

The relationship of clinical periodontal parameters and MMP-3 and TIMP-1 levels was analysed to assess the efficacy of PACNs in periodontal therapy. Concentrations of immunologic markers, MMP-3, and TIMP-1 in saliva were assessed at baseline and at 8 weeks following therapy.

For MMP-3/TIMP-1 detection, unstimulated saliva samples were collected using a Salivette (SARSTEDT AG and Co) saliva sample collection kit. Unstimulated saliva samples were collected at baseline and at 8 weeks following therapy (MINST or MINST + PACNs).

Immunologic investigation was performed by the same examiner (RB). Samples were centrifuged at 3500 rpm (2 min) and aliquoted, and a protease inhibitor cocktail (1 mg/mL) was added to each sample. All samples were stored at −80 °C and evaluated using enzyme-linked immunosorbent assay (ELISA), according to the manufacturer's instructions. MMP-3 and TIMP-1 concentration in saliva samples were examined using commercial ELISA kits (Elabscience) and a Multiskan Microplate Photometer (Thermo Fisher Scientific) at a 450-nm wavelength. Results were calculated using the standard curves formed by each assay for saliva and are given in pg/mL.

### Sample size

A sample size of 46 patients, 23 in each group (control and test), was sufficient to detect a clinically important difference in the mean PPD change from baseline in moderate sites at 8 weeks between the control and test groups using a 2-tailed *t* test for mean difference with 78.9% power and a 5% level(α=0.05).

### Randomisation

A total of 46 patients with periodontitis were randomised into 2 groups. RP performed the coding and randomisation of the participants in the treatment groups with a computer-generated randomisation table. At first, the patients were issued unique numbers from 1 to 46. After the coding procedure, a simple randomisation without restrictions was performed, and 2 sets of randomised numbers were generated (23 each). The allocation concealment was done by associating the first set of numbers to the control group (MINST) and the second set to the test group (MINST + PACNs).

### Blinding

During the MINST procedure, the clinician (periodontist EA) was not informed about the patients’ group allocation (MINST or MINST + PACNs). After the MINST procedure, the patients’ group assignments were revealed to the clinician by the examiner (RP), and accordingly the patients received the intended treatment.

From the beginning of the clinical trial, the patients were not informed which group they have been allocated to. The periodontal treatment was performed under local anesthesia in a sterile field (dental surgery face drapes were used) to eliminate the possibility for patients to monitor the process of the procedure. Thus, patients could not see or feel what type of treatment (MINST of MINST + PACNs) they received.

### Statistical methods

The effect of periodontal treatment was reflected by clinical periodontal measures (PPD, CAL, BOP, and PI) and 2 biochemical measures (MMP-3 and TIMP-1 concentrations in saliva). PPD, BOP, CAL, and PI were measured at 6 sites per tooth: mesiobuccal, buccal, distobuccal, distolingual, lingual, and mesiolingual. The clinical measurements of third molars were excluded from the study. Per-patient PPD and CAL of moderate pockets at baseline (and at 8 weeks) were obtained by averaging the PPDs and CALs in moderate sites for each patient at baseline (at 8 weeks). Similarly, per-patient PPD and CAL of deep pockets at baseline (and at 8 weeks) were obtained by averaging the PPDs and CALs in deep sites for each patient at baseline (and at 8 weeks). Per-patient PPD (and CAL) change from baseline in moderate (deep) pockets was obtained by averaging the PPD (and CAL) changes within each patient by baseline PPD (4–6 mm or ≥7 mm). Per-patient BOP (and per-patient PI) was obtained by calculating a percentage share of tooth sites with BOP (and plaque) for each patient and by classifying pockets by baseline PPD (4–6 mm or ≥7 mm).

Statistical analysis was performed with IBM SPSS 28 statistic software package (IBM Corp.). Shapiro–Wilk test was performed to assess whether clinical periodontal measures (per-patient PPD, CAL, BOP, and PI) and biochemical measures (MMP-3 and TIMP-1 concentrations in saliva) followed a normal distribution. Accordingly, if the data followed a normal distribution, a paired-samples *t* test was done to obtain before- and after-treatment comparisons within the groups. If the assumption of normality was violated, related-samples Wilcoxon signed rank test was done to obtain before- and after-treatment comparisons within the groups. The between-group comparisons of measures were determined by either independent-samples *t* test in case the specific measure followed a normal distribution or Mann–Whitney test in case the specific measure followed a non-normal distribution. The statistical significance level was considered at the .05 level.

Ethical approval was obtained from the Kaunas Regional Biomedical Research Ethics Committee (No. BE-2-38). All methods were carried out in accordance with relevant guidelines and regulations. All protocols were approved by the Regional Biomedical Research Ethics Committee.

## Results

### Participant flow

**Fig. 1:**
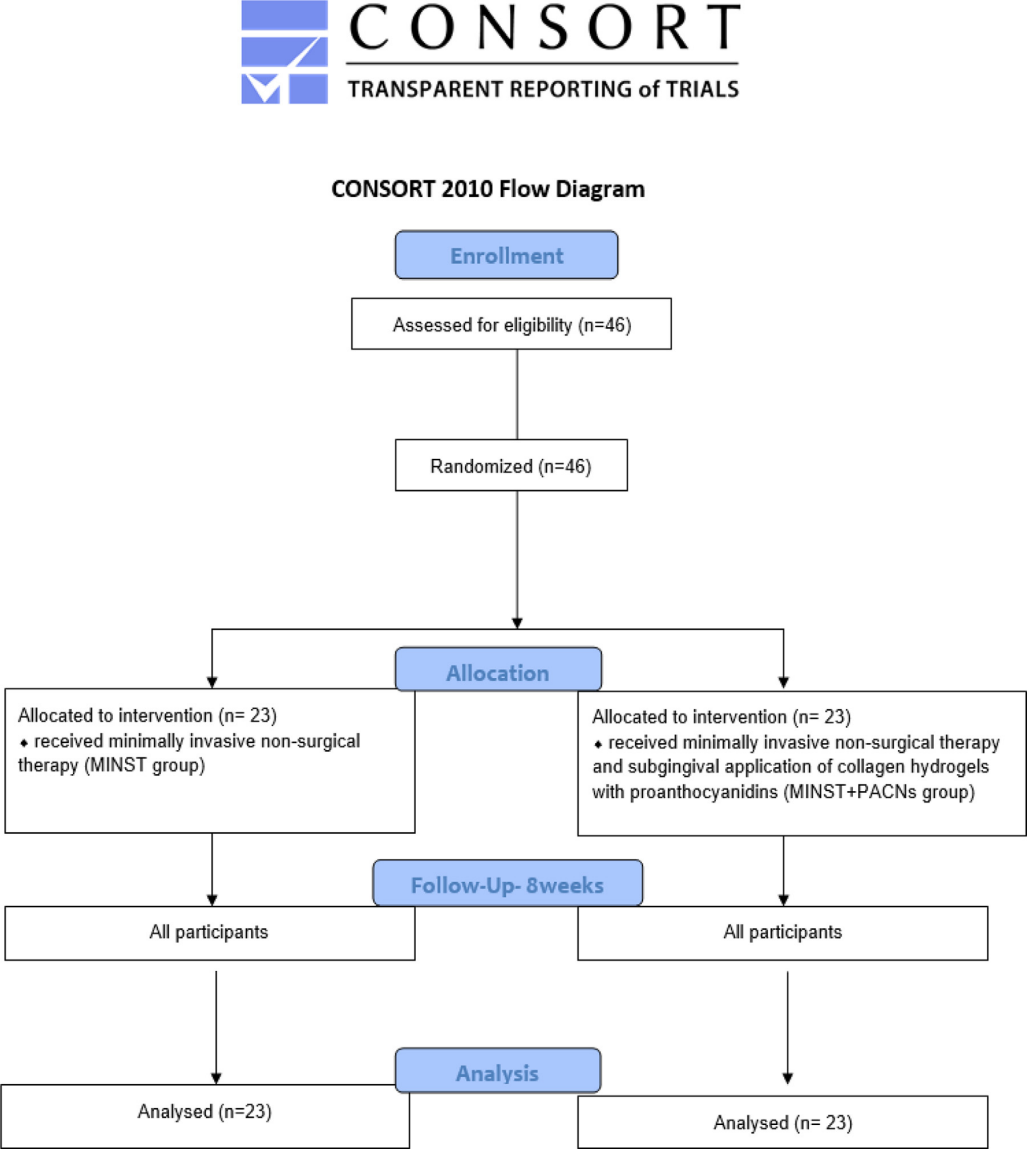
CONSORT flow diagram of participant recruitment.

Forty-six patients (stage of periodontitis III–IV) completed the study. Each treatment group (MINST or MINST +PACNs) consisted of 23 randomly selected patients. The number of participants is shown in the CONSORT flow diagram (Figure 1).

### Baseline characteristics

There were no statistically significant differences found between the 2 groups (MINST or MINST + PACNs) regarding age, gender, tobacco use (past or current), or periodontitis stage at the baseline visit (Table 1).

**Table 1:** Clinical and demographic characteristics of sample population at the baseline.

Characteristic	MINST	MINST + PACNs	*P* value
	n = 23	n = 23	
Age, y (mean ± SD)	46.3 ± 9.5	51.2 ± 1.5	.109^a^
Gender, n (%)			
Female	17 (73.9)	16 (69.6)	.743^b^
Male	6 (26.1)	7 (30.4)	
Tobacco use, n (%)			
Current use	8 (34.8)	7 (30.4)	.753^b^
No use	15 (65.2)	16 (69.6)	
Periodontitis stage, n (%)			
III stage	16 (69.6)	11 (47.8)	.134^b^
IV stage	7 (30.4)	12 (52.2)	

a Independent-samples *t* test.

b *χ*^2^ test. (Significant differences indicated in bold) MINST, minimally invasive nonsurgical therapy; PACN, proanthocyanidins.

### Effect on clinical parameters

#### Effect on probing depth

The mean per-patient PPD dynamics in moderate (baseline PPD 4–6 mm) and deep (baseline PPD ≥7 mm) pockets are presented in Table 2.

**Table 2:** Clinical characteristics of sample population at the baseline and after 8 weeks.

Characteristic	MINST			MINST + PACNs		
	n = 23			n = 23		
	Baseline	8 wk	*P* value	Baseline	8 wk	*P* value
CAL, mm (mean ± SD)						
Moderate pockets	6.2 ± 0.6	3.7 ± 0.5	<.001*	7.0 ± 0.7	4.0 ± 0.9	<.001*
Deep pockets	9.2 ± 1.0	5.8 ± 1.1	<.001*	9.4 ± 0.8	5.8 ± 1.0	<.001*
PPD, mm (mean ± SD)						
Moderate pockets	5.0 ± 0.3	2.1 ± 0.6	<.001*	5.5 ± 0.3	2.1 ± 0.5	<.001*
Deep pockets	7.7 ± 0.6	2.7 ± 1.2	<.001*	7.8 ± 0.5	2.7 ± 0.9	<.001*
% sites with PPD						
Shallow sites	-	81.4 ± 17.2	**<.001**‡	-	85.9 ± 12.4	**<.001**‡
Moderate pockets	81.0 ± 17.5	16.9 ± 14.4	**<.001**†	69.1 ± 19.8	13.4 ± 11.7	**<.001**†
Deep pockets	19.0 ± 17.5	1.7 ± 5.7	**<.001**†	30.9 ± 19.8	0.6 ± 1.7	**<.001**†
≥5 mm	79.7 ± 12.9	9.9 ± 12.0	**<.001**†	94.7 ± 8.8	6.6 ± 9.9	**<.001**†
≥6 mm	41.3 ± 22.9	3.8 ± 7.7	**<.001**†	68.6 ± 21.8	2.7 ± 5.5	**<.001**†
≥7 mm	19.0 ± 17.5	1.7 ± 5.7	**<.001**†	30.9 ± 19.8	0.6 ± 1.7	**<.001**†
≥8 mm	10.0 ± 12.2	0.5 ± 1.9	**.001**†	18.6 ± 15.8	0.0 ± 0.0	**<.001**†
≥9 mm	5.3 ± 10.7	0.1 ± 0.5	**.008**†	5.1 ± 6.9	0.0 ± 0.0	**.005**†
BOP, % sites (mean ± SD)						
Moderate pockets	91.4 ± 16.4	13.3 ± 18.2	**<.001**†	89.2 ± 24.5	22.9 ± 29.1	**<.001**†
Deep pockets	92.1 ± 24.3	17.2 ± 27.3	**<.001**†	91.2 ± 24.7	27.2 ± 32.2	**<.001**†
PI, % sites (mean ± SD)						
Moderate pockets	57.2 ± 37.0	10.6 ± 21.8	**<.001**†	69.5 ± 33.7	22.6 ± 33.6	**<.001**†
Deep pockets	65.1 ± 40.5	11.6 ± 26.8	**<.001**†	73.7 ± 36.8	28.8 ± 42.3	**.002**†

Significance level is .05 are shown in bold.

⁎ Paired-samples *t* test (α=0.05).

† Related-samples Wilcoxon signed rank test (α=0.05).

‡ One-sample *t* test, test value = 0 (α=0.05). BOP, bleeding on probing; CAL, clinical attachment level; MINST, minimally invasive nonsurgical therapy; PACN, proanthocyanidins; PI, plaque index; PPD, pocket probing depth.

The mean PPD for moderate pockets in the MINST group significantly differs from the mean PPD for moderate pockets in the MINST + PACNs group at baseline (*P* < .001; Table 2). Since the means showed statistically significant differences between the groups at baseline, further analysis was done by comparing the mean changes in PPD after 8 weeks from baseline. The conclusions of the current study were also drawn from the mean changes in PPD.

In both groups, the mean per-patient PPD of moderate and deep pockets reduced statistically significantly after 8 weeks compared to baseline. Specifically, after 8 weeks in the MINST group the mean per-patient PPD reduced from 5.0 (SD = 0.3) mm to 2.1 (SD = 0.6) mm in moderate pockets (*P* < .05) and from 7.7 (SD = 0.7) mm to 2.7 (SD = 1.2) mm in deep pockets (*P* < .05). The adjunctive application of PACNs to MINST after 8 weeks resulted in a mean per-patient PPD reduction from 5.5 (SD = 0.3) mm to 2.1 (SD = 0.5) mm in moderate pockets (*P* < .05) and from 7.8 (SD = 0.5) mm to 2.7 (SD = 0.9) mm in deep pockets (*P* < .05).

The comparison of mean per-patient change in PPD between the 2 treatment types revealed that on average the change in PPD was statistically significantly greater in moderate pockets in the MINST + PACNs group compared to the MINST group (Table 3).

**Table 3:** Effect of MINST and MINST + PACNs on change in clinical parameters after 8 weeks from baseline.

Reduction in characteristic at 8 wk from baseline	MINST	MINST + PACNs	*P* value
	n = 23	n = 23	
CAL, mm (mean ± SD)			
Moderate pockets	2.5 ± 0.6	3.1 ± 0.6	.002*
Deep pockets	3.4 ± 1.0	3.7 ± 1.0	.455*
PPD, mm (mean ± SD)			
Moderate pockets	2.9 ± 0.6	3.4 ± 0.6	.019*
Deep pockets	5.0 ± 1.1	5.1 ± 1.2	.805*
% sites with PPD			
Moderate pockets	64.2 ± 23.4	55.6 ± 26.8	.345†
Deep pockets	17.2 ± 17.2	30.3 ± 19.7	**.030**†
≥5 mm	69.8 ± 17.6	88.1 ± 11.2	**<.001**†
≥6 mm	37.4 ± 21.7	65.9 ± 23.0	**<.001**†
≥7 mm	17.2 ± 17.2	30.3 ± 19.7	**.030**†
≥8 mm	9.4 ± 11.7	18.6 ± 15.8	**.029**†
≥9 mm	5.2 ± 10.6	5.1 ± 6.9	.632†
BOP, % sites (mean ± SD)			
Moderate pockets	78.1 ± 28.5	66.2 ± 31.9	.168†
Deep pockets	74.8 ± 32.5	64.0 ± 33.8	.258†
PI, % sites (mean ± SD)			
Moderate pockets	46.6 ± 34.2	46.9 ± 43.9	.783†
Deep pockets	53.5 ± 39.7	44.9 ± 46.4	.663†

Significance level is .05 are shown in bold.

⁎ Independent-samples *t* test (α=0.05).

† Independent-samples Mann–Whitney test (α=0.05). BOP, bleeding on probing; CAL, clinical attachment level; MINST, minimally invasive nonsurgical therapy only; PACN, proanthocyanidins; PI, plaque index; PPD, pocket probing depth.

Specifically, in moderate pockets the application of adjunctive PACNs resulted in a 0.5-mm greater per-patient PPD reduction (*P* < .05) on average compared to MINST alone. However, in deep pockets no statistically significant differences were found in mean per-patient PPD between the 2 treatment modalities.

#### Effect on BOP

The BOP dynamics in moderate and deep pockets are presented in Table 2. Both treatment modes had a positive effect on reducing the inflammation in moderate and deep pockets, as the per-patient BOP measures dropped after 8 weeks compared to baseline. The reduction in per-patient BOP was statistically significant in both groups. However, the comparison of per-patient BOP changes in moderate and deep pockets between the groups did not show any significant differences (Table 3).

### Effect on salivary biomarkers

**Fig. 2:**
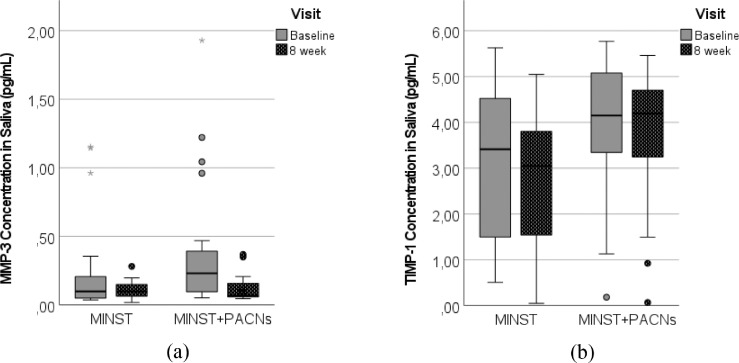
Effect of specific periodontal treatment on MMP-3 (A) and TIMP-1 (B) concentrations in saliva in pg/mL by group and visit. Periodontal treatment groups: MINST and MINST + PACNs. The boxplots of MMP-3 and TIMP-1 concentration in saliva are presented. #*P* < .05 vs MMP-3 concentration in saliva at the baseline, related-samples Wilcoxon signed ranks test. MINST, minimally invasive nonsurgical therapy; MMP-3, matrix metalloproteinase-3; PACN, proanthocyanidins; TIMP-1, tissue inhibitor of matrix metalloproteinase-1.

A goal of this study was to determine whether MMP-3 might be considered as a biomarker of periodontitis. A total of 46 individuals with periodontitis were included in the study. The effects of MINST and MINST + PACNs treatment on MMP-3 and TIMP-1 levels in saliva are presented in Figure 2A and 2B.

The MMP-3 concentration in the MINST + PACNs group reduced statistically significantly after treatment when compared to baseline (Figure 2A). However, the MMP-3 concentration in the MINST group did not differ statistically significantly after treatment when compared to baseline. No statistically significant improvements were found in TIMP-1 concentration in saliva for either group after 8 weeks compared to the baseline level (Figure 2B).

**Fig. 3:**
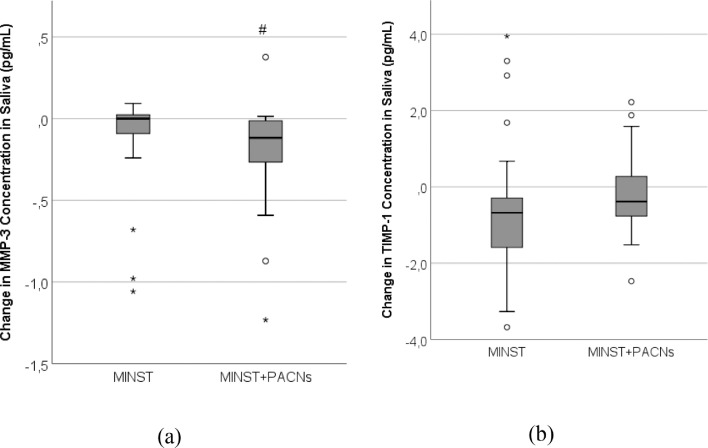
The effect of specific periodontal treatment on change in MMP-3 (A) and TIMP-1 (B) concentrations in saliva by group. Periodontal treatment groups: MINST and MINST + PACNs. The boxplots of changes in concentrations of MMP-3 and TIMP-1 in saliva are presented. #*P* < .05 vs the change in MMP-3 concentration in saliva in MINST group, Mann–Whitney test. MINST, minimally invasive nonsurgical therapy; MMP-3, matrix metalloproteinase-3; PACN, proanthocyanidins; TIMP-1, tissue inhibitor of matrix metalloproteinase-1.

The effect of MINST and MINST + PACNs treatment on change in MMP-3 and TIMP-1 concentrations in saliva is presented in Figure 3A and 3B. Figure 3A shows that MMP-3 values in saliva reduced in the MINST + PACNs group more than in the MINST group, and this result is statistically significant. The analysis of change in TIMP-1 concentration revealed that no statistically significant improvements were found in the change in TIMP-1 concentration in saliva between the groups (Figure 3B).

No adverse effects were recorded over the whole study period. In addition, we used the economic assumptions and calculated cost-effectiveness of PACNs as a product. Our conclusion is that adjunctive materials (eg, chlorhexidine chips, tetracycline fibers) are similarly cost-effective as PACNs.^30^

## Discussion

The current study estimated the effects of PACNs on the treatment of generalised periodontitis by analysing clinical and immunologic parameters. The results showed that adjunctive use of PACNs combined with MINST promoted additional beneficial effects on clinical and immunologic parameters. Adjunctive use of PACNs showed statistically significantly higher PPD reduction in moderate periodontal pockets.

To the authors’ knowledge, this clinical study is the first randomised controlled clinical trial to evaluate the efficacy of locally derived PACNs in the treatment of generalised periodontitis. Until now, only 2 clinical studies have reported the investigation of clinical effects of systemically administered PACN nutritional supplements on periodontal health.^19^^,^^31^ In one study, systemically administered PACNs decreased the culturable bacteria counts and resulted in statistically significant improvement of clinical measurements (PPD, gingival bleeding index). ^31^ In another study, the nutritional supplement made of PACNs induced an improvement in periodontal health (lower gingival bleeding index and the Silness and Löe Index in the experimental group).^19^

Clinical research investigating nonsurgical periodontal therapy and adjunctive therapy agreed that locally derived adjuncts showed an additional benefit in clinical parameters.^13^ Research showed that adjunctive use of local antibiotics in nonsurgical periodontal therapy demonstrated statistically significant PPD reduction by 0.37 mm when compared with control groups.^32^ According to recent research, adjunctive use of locally derived sodium hypochlorite gel in MINST resulted in additional reduction of PPD by 0.5 mm (*P* = .001).^33^ Similarly, the current study showed that adjunctive use of PACNs resulted in additional PPD reduction in moderate periodontal pockets by 0.5 mm (*P* < .05) on average compared to MINST alone. According to the literature^34^ and the current trial, it can be concluded that nonsurgical periodontal therapy should still be the first choice for initial treatment of deep periodontal pockets due to the high PPD reduction in deep pockets following treatment.

The results of the current study showed no statistically significant differences in BOP values after treatment between the groups. Collagen hydrogels release active substance (PACNs) and dissolve in periodontal pockets quite rapidly (resorption rate usually takes ≥4 weeks).^35^ Regardless of the treatment group (MINST or MINST + PACNs), later stages of the healing process may happen similarly and therefore result in similar outcomes (BOP values). This study implies that both treatment strategies may help to reduce inflammation and improve periodontal health in terms of BOP values.

MMPs are one of the most important proteinases, responsible for hydrolysis of components of the extracellular matrix, tissue remodeling, wound healing, and angiogenesis. MMPs can degrade almost all compounds of extracellular matrix and basal membrane, and their redundant activity can lead to periodontal tissue degradation.^36^ Although the mechanisms are not very clear, MMPs are usually known as tissue-remodelling enzymes that are activated during an inflammatory response.^37^ The decision to investigate MMP-3 and TIMP-1 levels was chosen according to similar clinical studies. The authors reported that higher MMP-3 and TIMP-1 levels correlated positively with clinical parameters and were related to significantly greater risk of progression of periodontal disease.^4^^,^^38^, ^39^, ^40^ Further analysis of MMP-3 and TIMP-1 levels was included in the current clinical trial of nonsurgical periodontal treatment with PACNs.

As mentioned earlier, preclinical studies have shown high antibacterial and anti-inflammatory capacities of PACNs.^20^ In vitro studies with PACNs show that they inhibit the production of MMPs.^41^, ^42^, ^43^ The results of preclinical studies reflect the conclusions of the current clinical study: MMP-3 values in saliva reduced in the MINST + PACNs group more than in the MINST group.

Our study focused on the analysis of MMP and TIMP proportions to predetermine the level of periodontal tissue destruction in patients with periodontitis. More than 2 decades ago, clinical trials suggested that MMP-3 and TIMP-1 levels may be an additional parameter for periodontal health evaluation.^44^ Research reported that relatively low MMP-3 levels in gingival crevicular fluid raise the question of whether there is enough sensitivity and specificity for this biochemical marker to evaluate periodontal disease progression.^45^ However, recent research has shown that MMP-3 and TIMP-1 values may be used as an indicator in the evaluation of periodontal health status.^39^^,^^46^, ^47^, ^48^ This clinical trial showed that MMP-3 levels in saliva are related to the severity of chronic periodontal infection.

In addition, our study analysed MMP-3 and TIMP-1 changes in relation to nonsurgical periodontal therapy (MINST and MINST + PACNs). Results showed that MMP-3 values in saliva were reduced significantly more in the MINST + PACNs group than in the MINST group. This observation may suggest that additional use of PACNs in periodontal treatment decreases MMP-3 concentrations in saliva more than MINST alone. However, the analysis revealed that no statistically significant differences were observed in the change of TIMP-1 concentration between the groups.

There are some limitations of this study. Because the clinical study was designed as a short-term trial (clinical reevaluation 8 weeks after the treatment), it is necessary to determine whether there were any long-term clinical results (at least 6 months after the treatment)^49^ of PACNs in MINST. The short duration of the observation period of this study (8 weeks) should be noted as a limitation. At 8 weeks posttreatment, when the periodontal reevaluation is performed, further instructions according to the EFP S3 level clinical practice guidelines are followed.^50^ Regardless of the blinding used in this trial, the study did not use placebo treatment to conceal allocated treatment and to reduce bias. Moreover, smoking status was not defined as an exclusion criterion. The measurement of CAL was not included in the protocol of the current study but we agree that clinical attachment level could be useful reflective measurement of periodontal disease severity.

## Conclusions

Within the limits of this study, adjunctive use of PACNs with MINST has been shown to result in additional PPD reduction and CAL gain in moderate periodontal pockets compared to MINST alone. Immunologic investigation showed that additional use of PACNs in MINST reduced MMP-3 concentrations in saliva more than MINST alone.

## Author contributions

All authors collaborated to generate a plan and perform all the necessary experimental procedures. Rasa Pušinskaitė performed the statistical analysis. Rasa Banienė, Evelina Alkimavičienė, and Rasa Pušinskaitė analysed and summarised the experimental results. Evelina Alkimavičienė and Rasa Pušinskaitė wrote the paper. Nijolė Savickienė, Nomeda Basevičienė, Rasa Banienė, and Ingrida Marija Pacauskienė performed the interpretation of data and critically amended the manuscript. All authors approved the final version to be published.

## Conflict of interest

None disclosed.
